# Antibiotic-induced stress responses in Gram-negative bacteria and their role in antibiotic resistance

**DOI:** 10.1093/jac/dkaf068

**Published:** 2025-03-07

**Authors:** Chanté Brand, Mae Newton-Foot, Melanie Grobbelaar, Andrew Whitelaw

**Affiliations:** Division of Medical Microbiology and Immunology, Department of Pathology, Faculty of Medicine and Health Sciences, Stellenbosch University, Cape Town, South Africa; Division of Medical Microbiology and Immunology, Department of Pathology, Faculty of Medicine and Health Sciences, Stellenbosch University, Cape Town, South Africa; National Health Laboratory Service, Tygerberg Hospital, Cape Town, South Africa; South African Medical Research Council Centre for Tuberculosis Research, Division of Molecular Biology and Human Genetics, Department of Biomedical Sciences, Faculty of Health Sciences, Stellenbosch University, Cape Town, South Africa; Division of Medical Microbiology and Immunology, Department of Pathology, Faculty of Medicine and Health Sciences, Stellenbosch University, Cape Town, South Africa; National Health Laboratory Service, Tygerberg Hospital, Cape Town, South Africa

## Abstract

Bacteria adapt to changes in their natural environment through a network of stress responses that enable them to alter their gene expression to survive in the presence of stressors, including antibiotics. These stress responses can be specific to the type of stress and the general stress response can be induced in parallel as a backup mechanism. In Gram-negative bacteria, various envelope stress responses are induced upon exposure to antibiotics that cause damage to the cell envelope or result in accumulation of toxic metabolic by-products, while the heat shock response is induced by antibiotics that cause misfolding or accumulation of protein aggregates. Antibiotics that result in the production of reactive oxygen species (ROS) induce the oxidative stress response and those that cause DNA damage, directly and through ROS production, induce the SOS response. These responses regulate the expression of various proteins that work to repair the damage that has been caused by antibiotic exposure. They can contribute to antibiotic resistance by refolding, degrading or removing misfolded proteins and other toxic metabolic by-products, including removal of the antibiotics themselves, or by mutagenic DNA repair. This review summarizes the stress responses induced by exposure to various antibiotics, highlighting their interconnected nature, as well the roles they play in antibiotic resistance, most commonly through the upregulation of efflux pumps. This can be useful for future investigations targeting these responses to combat antibiotic-resistant Gram-negative bacterial infections.

## Introduction

Gram-negative bacteria are a heterogeneous group of organisms, some of which are found in the environment, some are human or animal commensals, and some are opportunistic pathogens able to case a variety of infections. The survival of these organisms depends on their ability to overcome the many stressors that they encounter in their natural environment, such as changes in pH, temperature or osmolarity, and exposure to harmful substances like antibiotics. They do so with the help of a network of stress responses that enable them to modify their gene expression to adapt to new environmental conditions.^1^ These include a range of adaptive stress responses to overcome specific stressors, as well as the general stress response that promotes survival in response to a variety of stressors.^2,3^

This review provides an overview of stress responses that are induced by antibiotics which are commonly used to treat Gram-negative bacterial infections, and how this leads to antibiotic resistance. Understanding the intricate processes involved in antibiotic survival could lead to novel developments to combat the threat that antibiotic resistance poses to human health.

## Envelope stress responses

The cell envelope is a crucial component of the Gram-negative bacterial cell, consisting of inner and outer membranes (OMs) that control the movement of molecules into and out of the cell, as well as a thin peptidoglycan layer in the periplasmic space which provides structural integrity.^4^ Any threat to the cell envelope that results in the misfolding of OM proteins (OMPs) and lipopolysaccharides (LPS) is overcome by the envelope stress responses (ESRs). Therefore, ESR regulons can be induced by antibiotics which damage the bacterial cell envelope, such as β-lactams that inhibit cell wall synthesis, aminoglycosides that result in protein misfolding, and polymyxins that disrupt the OM by interacting with LPS.

**Figure 1.**
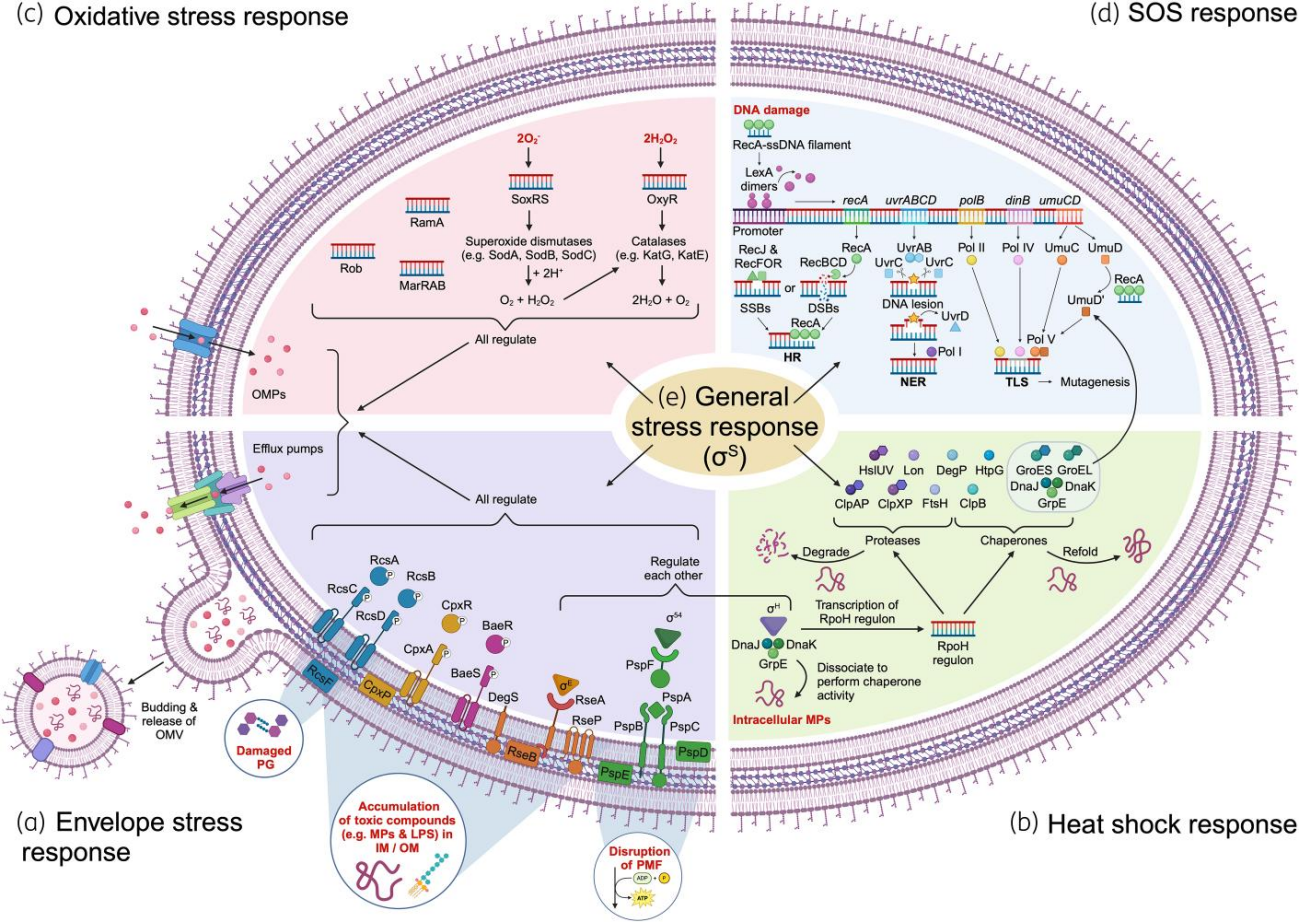
Bacterial stress responses induced by antibiotics. (a) Oxidative stress response, (b) SOS response, (c) ESR, (d) Heat shock response, (e) General stress response. Stressors are indicated in bold red text. DSBs, double strand breaks; HR, homologous recombination; IM, inner membrane; LPS, lipopolysaccharides; MPs, misfolded proteins; NER, nucleotide excision repair; OM, outer membrane; OMPs, outer membrane proteins; OMV, outer membrane vesicle; PG, peptidoglycan; SSB, single strand breaks; TLS, translesion synthesis. Created with BioRender.com.

Five main ESRs have been described in Gram-negative bacteria: the Psp (phage shock protein), Cpx (conjugative pilus expression), σ^E^ (sigma E), Bae (bacterial adaptive) and Rcs (regulator of capsule synthesis) systems. The production of OM vesicles (OMVs) has additionally been described as a complementary general ESR mechanism.^5^ Once induced, these ESRs regulate transcription of numerous adaptive proteins, such as those involved in envelope biogenesis, degradation or refolding of misfolded proteins, entry and export of toxic compounds, virulence, motility, capsule production, and biofilm formation,^6–10^ to repair the damage caused and maintain the integrity of the cell envelope.^11^ This includes regulating entry and export of antibiotics, and many porins and efflux flumps involved in antibiotic transport are regulated by multiple ESRs (Figure 1).

### The Cpx response

The Cpx two-component system (TCS) is widely conserved in Gram-negative bacteria^12^ and responds to stress at the inner membrane (IM) of the cell envelope.^13,14^ Stresses such as changes in the lipid composition or misfolded proteins that aggregate in the IM result in the inactivation of the inhibitor, CpxP, and trigger the phosphorylation of the response regulator, CpxR, by the sensor histidine kinase, CpxA.^15,16^ This changes the expression of hundreds of genes involved in various cellular functions within the bacterial envelope, including cell wall synthesis, protein folding and transport, as well as antibiotic resistance.^14^

The Cpx response is commonly induced by β-lactams in *Escherichia coli*, *Klebsiella aerogenes and Salmonella enterica* serovar Typhimurium, as well as fosfomycin in *E. coli* and aminoglycosides in *S*. Typhimurium (Table 1). Interestingly, survival in the presence of β-lactams and aminoglycosides has been demonstrated to be affected by the extent of induction of the Cpx response.^17,96^ Through genetic manipulation, it was found that moderate induction of the Cpx response in *E. coli* results in tolerance to β-lactams due to strengthening of the cell wall.^17^ In contrast, Cpx induction above a certain threshold results in defects in cell growth, division and shape, and therefore increased susceptibility to β-lactams.^17^

**Table 1.** Antibiotic induction of ESRs and their role in antibiotic resistance

Regulon/response	Organism^a^	Antibiotic inducer(s)	Antibiotics to which resistance conferred	Resistance mechanisms	
				Up-regulated	Down-regulated
Cpx	*E. coli* ^b^	Cephalosporins, fluoroquinolones, fosfomycin, penicillins^4,17,18^	Aminoglycosides, cephalosporins, fosfomycin, fluoroquinolones, monobactams, penicillins^4,17,19–23^	AcrD, MdtA^24,25^	OmpF, GlpT, UhpT^18,25,26^
	*K. aerogenes*	Carbapenems^27^	Aminoglycosides, carbapenems, cephalosporins, fosfomycin^27^	AmpC, AcrD, TolC^27,28^	Omp35, Omp36^27,28^
	*K. pneumoniae*	NR	Aminoglycosides, carbapenems, cephalosporins, chlorhexidine, macrolides, polymyxins, rifamycins tetracyclines^29,30^	AcrD, KpnEF^29,30^	OmpK36^30^
	*P. aeruginosa*	NR	Cephalosporins, fluoroquinolones, monobactams^31^	MexAB-OprM^31^	NR
	*S*. Typhimurium	Aminoglycosides, cephalosporins^23,32,33^	Aminoglycosides, cephalosporins, macrolides, tetracyclines^21,23,32,33^	AcrD, MdtA, STM1530, STM3031^21,32,33^	OmpC, OmpD, OmpF OmpW^21,23,32,33^
	*V. cholerae* ^b^	NR	Penicillins^34^ Aminoglycosides^35^	VexAB, VexGH^34^	NR
BaeR	*A. baumannii*	Tigecycline^36,37^	Tigecycline^37^	AdeA, AdeB^37^, AdeIJK, MacAB-TolC^36^	
	*E. coli*	Cephalosporins, penicillins^4^	Cephalosporins, monobactams, novobiocin^19,22,24,38–41^	AcrD, MdtABC^19,22,24,25,38,39,41–45^	OmpC, OmpF, OmpW, OmpA OmpX^41^
	*S*. Typhimurium	NR	Aminoglycosides, cephalosporins, macrolides, novobiocin, tetracyclines^21,42^	AcrD, MdtABC, STM1530, STM3031, TolC^21,42,45–47^	OmpD^21^, OmpW^47^
Rcs	*E. coli*	Cephalosporins, penicillins, nitrofurantoin, polymyxins^4,48–50^	Aminoglycosides, carbapenems, cephalosporins, fosfomycin, monobactams, penicillins^24,40,51^	AcrD, MacA, MdtA^24^	FlhDC^48,52^
	*K. pneumoniae*	Polymyxins^53^	Polymyxins^53^	NR	NR
	*S*. Typhimurium	Polymyxins^54,55^	Polymyxins^54,56^	NR	NR
	*S. marcescens*	Cephalosporins, polymyxins,^57^	NR	NR	NR
Sigma E	*E. coli*	Carbapenems, cephalosporins, penicillins^4,58^	Polymyxins, rifamycins^59,60^	SmpA^60^	OmpA, OmpC, OmpN, OmpW^58,61,62^
	*K. pneumoniae*	Carbapenems^63^	Carbapenems, cephalosporins^63^	NR	OmpK35, OmpK36^63^
	*S*. Typhimurium	NR	Polymyxins, rifamycins^64,65^	SmpA^65^	OmpA, OmpC, OmpD, OmpF, OmpW^66^
	*P. aeruginosa*	Carbapenems, chlorhexidine, penicillins, polymyxins^67–74^	Carbapenems, cephalosporins, chlorhexidine, polymyxins^67,70,74^	AmpC, MexCD-OprJ, OmlA (SmpA homologue)^67,68,70–75^	NR
	*Vibrio spp.*	Polymyxins (*V. cholerae*)^76^	Polymyxins (*V. parahaemolyticus*)^77^	NR	NR
Psp	*E. coli*	Penicillins, fluoroquinolones^50^	Cephalosporins, monobactams, carbapenems, fluoroquinolones^40,78,79^	NR	NR
	*K. pneumoniae*	Carbapenems^80^	NR	NR	NR
Outer membrane vesicles	*A. baumannii*	Carbapenems, cephalosporins^81–83^	Carbapenems, penicillins, penicillins + BLIs^84^	OXA-23, OXA-24, OXA-58^82,84^	NR
	*Bacteroides* spp.^85^	NR	Cephalosporins^85^	CepA β-lactamase^85^	
	*E. coli*	Carbapenems, fosfomycin, fluoroquinolones, polymyxins^5,86,87^	Cephalosporins, penicillins, polymyxins^5,88^	OmpW, OmpC, OmpF, CTX-M-1 (within OMVs)^88^	TolC (within OMVs)^88^
	*H. pylori*	NR	Fluoroquinolones, macrolides^89^	NR	NR
	*M. catarrhalis*	NR	Penicillins^90^	Bro-1, Bro-2^90^	NR
	*P. aeruginosa*	Fluoroquinolones, penicillins, polymyxins^69,91,92^	NR	Unspecified β-lactamase, possibly inducible class C^91^	NR
	*S. maltophilia*	Carbapenems, fluoroquinolones, penicillins^93–95^	Carbapenems, penicillins^93,94^	L1 MBL, L2 SBL, Smlt0387, Smlt0184, unspecified β-lactamase^93,94^	NR

BLI, β-lactam + β-lactamase inhibitor combination; MBL, metallo-β-lactamase; NR, not reported (to the best of our knowledge); SBL, serine-β-lactamase.

^a^Full organism names: *Acinetobacter baumannii*, *Escherichia coli*, *Klebsiella aerogenes*, *Klebsiella pneumoniae*, *Moraxella catarrhalis*, *Pseudomonas aeruginosa*, *Salmonella enterica* serovar Typhimurium, *Stenotrophomonas maltophila*, *Vibrio cholerae*.

^b^Including the Zra TCS in the CpxP superfamily.

The opposite is suggested for aminoglycosides. It is thought that lethal intracellular levels of aminoglycosides can be reached before Cpx induction is sufficient to confer resistance.^96^ However, upon constitutive activation of Cpx, the sustained high levels of stress-fighting proteins are able to prevent lethal damage and increase survival.^96^ A combination of these models could explain why hyperactivation of the Cpx response in *E. coli* has been demonstrated to confer resistance to aminoglycosides but not to β-lactams^96^ or to confer only low-level β-lactam resistance.^19^ Bacteria therefore seem to fine-tune the regulation of Cpx to respond to specific stressors. The expression level of *cpxRA* has been found to change over time in *Citrobacter rodentium* during infection of mice, with higher levels during initial stages of infection.^97^

The mechanisms through which the Cpx response has been linked with resistance to β-lactams and aminoglycosides include upregulation of the MdtABC, AcrD and TolC efflux pump components and downregulation of the OmpC and OmpF porins (Table 1). Individual deletion of AcrD, MdtABC and TolC was shown to not affect the Cpx-mediated resistance to these agents.^24,32,96,98^ Stress responses are interconnected and deletion of one regulon member could result in upregulation of another to compensate and promote survival. MdtABC and AcrD are components of multidrug resistance (MDR) efflux pumps of the resistance-nodulation-cell division (RND) family transporters and are co-ordinately regulated together with other members of their respective efflux pumps.^99,100^ It has consequently been suggested that no known Cpx-regulon member contributes to aminoglycoside resistance on its own, but that the combined actions of multiple members might be required, although it is possible that one may be sufficient.^96,98^

The Zra TCS, which shares structural and functional similarities with Cpx,^20^ has also been linked with variable effects on resistance to β-lactams and aminoglycosides. Deletion of *zraP* (similar to *cpxP*) in *E. coli* has been reported to increase sensitivity to certain aminoglycoside and β-lactam antibiotics (capreomycin and amoxicillin, respectively) but decrease sensitivity to other aminoglycosides (amikacin and gentamicin) and β-lactams (cephalotin and cefuroxime).^20^ The authors suggested interconnectivity of extracytoplasmic stress responses as a reason for this. Both Zra and Cpx have also been reported to play a role in aminoglycoside tolerance in *Vibrio cholerae*.^35^

Cpx has also been implicated in fosfomycin resistance in *E. coli* by downregulating the GlpT and UhpT transporters which are required for fosfomycin uptake.^18^ Various species-specific multidrug efflux pumps such as KpnEF in *Klebsiella pneumoniae*,^29^ VexAB and VexGH in *V. cholerae*^34^ and MexAB-OprM in *Pseudomonas aeruginosa*^31^ are also regulated by the Cpx system (Table 1).

### The Bae response

The Bae pathway is a classical TCS, consisting of an inner-membrane-bound sensor histidine kinase, BaeS and a cytoplasmic response regulator, BaeR.^101^ The BaeR regulon of *E. coli* consists of the *acrD* gene, the *spy* gene (encoding a periplasmic chaperone that is also regulated by CpxAR), and the *mdtABCD-baeSR* operon.^101^ The latter encodes the BaeSR TCS proteins as well as components of the RND efflux pump, MdtABC-TolC, and the major facilitator superfamily (MFS) efflux pump, MdtD.^38,39,101^ The *mdtABCD-baeSR* operons of various well-known Gram-negative human pathogens are predicted to be identical (*S. enterica*, *Shigella* spp., *Yersinia* spp.) or similar (*K. pneumoniae*, *Enterobacter cloacae*, *Proteus mirabilis*) to that of *E. coli*.^9^

The main role of this ESR is to respond to specific envelope-damaging toxic compounds that are substrates of MdtABC by upregulating this efflux pump and thereby restoring envelope homeostasis by discarding these compounds.^9^ The BaeSR response has been reported to be induced by β-lactams, and to play a role in resistance to these and other agents (Table 1). This resistance is likely due to BaeR-mediated upregulation of AcrD, MdtABC and TolC, as well as downregulation of various OMPs (Table 1).

Upregulation of both AcrD and MdtABC is needed to result in BaeR-mediated antibiotic resistance.^19,24^ Furthermore, although TolC is up-regulated by BaeR to a lesser extent than AcrD or MdtABC, deletion of TolC reduced BaeR-mediated antibiotic resistance more than deletion of AcrD or MdtABC.^42^ This emphasizes the need for fine-tuned regulation of the TolC component of these efflux pumps to confer resistance. This could also explain why without TolC, Bae-mediated increased expression of certain individual components of these RND efflux pumps alone is not always sufficient to result in resistance to aminoglycosides.^21,24,39,46,96,98^

In *Acinetobacter baumannii*, another set of species-specific RND efflux pumps, AdeAB and AdeIJK, as well as the ABC transporter, MacAB-TolC, are regulated by tigecycline-mediated activation of BaeSR, resulting in tigecycline resistance.^36,102^

### The Rcs phosphorelay

The Rcs ESR senses damage to OM LPS^48^ and peptidoglycan.^4^ As its name suggests, it regulates the expression of capsular polysaccharide biosynthetic genes, which are involved in virulence as well as biofilm formation.^10,53,54,56,103^ The Rcs regulon encodes a multicomponent phosphorelay, involving a classic TCS consisting of the signal histidine kinase, RcsC, and the response regulator, RcsB, in addition to a phosphotransfer protein, RcsD, an OM sensory component, RcsF and auxiliary regulators, such as RcsA, which can act together with RcsB.^104,105^

This system is widespread among Gram-negative bacteria^105,106^ and has been reported to be induced by β-lactams, nitrofurantoin and polymyxin B (PMB) and to increase survival in the presence of these and other cell wall damaging antibiotics (Table 1). Interestingly, the Rcs system has also been shown to confer low-level resistance to kanamycin,^24^ but not to other protein synthesis inhibitors such as tetracycline and streptomycin.^51^

Furthermore, sub-inhibitory concentrations of PMB have been shown to strongly activate the Rcs system in *Serratia marcescens*, even though the test strain displayed high-level resistance to this agent.^57^ The same was shown for vancomycin,^57^ which is not used to treat Gram-negatives due to its inability to penetrate the OM of these organisms and reach its peptidoglycan target, resulting in high-level intrinsic resistance. Nevertheless, Gram-negative bacteria may be exposed to vancomycin in the environment or during the treatment of Gram-positive infections. It is therefore interesting to see that, even though they cannot prevent growth, these antibiotics are still able to disrupt the cell envelope and elicit a stress response.

The mechanism by which the Rcs phosphorelay contributes to antibiotic resistance is not well understood. Rcs was found to up-regulate the macrolide export protein, MacA, but did not affect resistance to the macrolide antibiotic, erythromycin.^24^ It has also been reported to up-regulate expression of AcrD and MdtA in *E. coli*, albeit to a low extent, although resistance is thought to be conferred by mechanisms other than drug exporters.^24^ Increased survival in the presence of antibiotics could additionally be attributed to the decreased expression of Rcs-regulated genes involved in flagellar synthesis, inhibiting motility and resulting in biofilm formation.^52,107,108^

Activation of the Rcs system has been found to be dependent on activation of Cpx, but not vice versa and the simultaneous action of both regulons is thought to be required to fully regulate certain cellular functions, such as inhibiting motility.^52^ Therefore, similarly to the other ESRs, Rcs induction may not be able to promote survival in the presence of antibiotics on its own.

### The sigma E response

The extracytoplasmic sigma factor (σ^E^ or σ^24^) stress response system senses misfolded OMPs and LPS within the OM and periplasmic space.^109^ These inducing cues trigger a signal transduction cascade involving the regulatory proteins, RseA and RseB, the IM proteases, DegS and RseP, and the cytoplasmic protease, ClpXP. This eventually results in activation of the σ^E^ transcription factor, encoded by *rpoE*, and transcription of the σ^E^ regulon members.^109,110^ This includes upregulation of chaperones and proteases to refold or degrade misfolded proteins, proteins involved in the biosynthesis and transport of LPSs, phospholipids, and OMPs to restore OM integrity.^109–111^

Expression of these and other σ^E^ regulon members has been shown to be induced upon exposure to β-lactams and linked to β-lactam resistance through decreased OMP expression and induction of the AmpC β-lactamase (Table 1). σ^E^ is also induced upon exposure to polymyxins and plays a role in survival in its presence.^59,64,67–69,77,112^ This has been attributed to the MDR efflux pump MexCD-OprJ in *P. aeruginosa*, which is regulated by its σ^E^ homologue, AlgU. MexCD-OprJ is also induced by chlorhexidine^68^ and has been linked to tolerance to both polymyxins and chlorhexidine in *P. aeruginosa* biofilms.^70^

Sigma factors like σ^E^, which direct RNA polymerase to specific promoters, do not directly carry out negative regulation. However, they induce expression of small RNAs (sRNAs) such as MicA, MicF, MicC and RybB, which negatively control the expression of various OMPs.^66,111,113^ These sRNAs, along with their OMP targets, are conserved across various Gram-negative species.^61,66,111^

### The Psp response

The Psp ESR, is activated by disruptions of IM integrity,^114^ including the dissipation of the proton motive force.^115^ Inducing signals are sensed by either or both of the cytoplasmic membrane proteins, PspB and PspC, which then bind to PspA to release it from PspF. This allows PspF to activate transcription of the *pspABCDE* operon, which is dependent on the alternative sigma factor σ^54^ (also known as σ^N^).^116^ The *psp* operon is strongly induced in response to infection by filamentous bacteriophages, ethanol exposure, as well as heat and hyperosmotic shock.^117^ Additionally, members of this operon have been reported to be induced by β-lactams and fluoroquinolones, and to play a role in survival in the presence of these agents (Table 1). The role of this ESR in response to antibiotics is not widely studied and the exact mechanism of its contribution to resistance is unclear, but it is thought to play a role in the formation of persisters.^78^

### Outer membrane vesicles

The production of OMVs is independent of the other ESR systems described above and provides a complementary mechanism of discarding misfolded proteins or foreign molecules that cause stress at the cell envelope.^5^ Throughout the course of normal growth in Gram-negative bacteria, these vesicles are formed by budding of the OM, trapping unwanted periplasmic and OM components inside and expelling them into the environment^5^ (Figure 1a). Increased OMV production in response to β-lactams, fluoroquinolones, polymyxins, fosfomycin and chloramphenicol, and subsequent increased survival in the presence of some of these agents, has been reported across a wide range of Gram-negative bacteria (Table 1).

Since they originate from the cell envelope, OMVs contain the usual envelope constituents such as LPS, OMPs and efflux pumps.^118^ They can therefore also contain β-lactamases which are located in the periplasmic space in Gram-negative bacteria (if encoded by the organism) and promote survival in the presence of β-lactam antibiotics.^81,82,85,90,91,93,94^ Proteomic analysis revealed increased abundance of OMPs and β-lactamases such as CTX-M, and decreased abundance of TolC in OMVs from β-lactam resistant *E. coli* strains compared with that of β-lactam susceptible strains.^88^ It is thought that these OMVs take up, retain and hydrolyze β-lactams through these mechanisms before β-lactams are able to enter the bacterial cell and reach their PBP targets.^88^

Moreover, secreted OMVs can adhere to and be internalized by other bacterial cells in the environment, resulting in the transfer of their contents.^119^ Therefore, OMVs have been linked with intra- and interspecies transfer of β-lactamases, across various Gram-negative bacterial species.^85,88,90,93,94^ Since OMVs may also contain mobile genetic elements, including those encoding β-lactamase genes, they may also mediate the transfer of β-lactamase genes.^84,119^

As described earlier with Cpx induction in response to β-lactams, there is thought to be an optimal level of OMV induction in response to antimicrobial peptides such as PMB.^120^ It was found that lower amounts of OMVs confer immediate protection against these agents, while allowing for the development of adaptive resistance. On the other hand, higher amounts of OMVs also result in immediate increased survival but prevents acquisition of long-term adaptive resistance to PMB.^120^ OMVs are also important components of Gram-negative biofilm matrices and are thought to protect biofilm cells from certain antibiotics by absorbing these agents and reducing the concentration that reaches the cells.^121^

Subinhibitory antibiotic exposure was also reported to affect the speed and distance of movement of OMVs within the cell membrane.^86^ This is thought to promote communication and horizontal gene transfer (HGT) between cells in close proximity, as well as changes in biofilm architecture, in an attempt to modulate stress.^86^

## Heat shock response

Bacteria respond to heat stress by upregulating the synthesis of heat shock proteins (HSPs) which combat the deleterious effects of protein misfolding and aggregation that result from exposure to elevated temperatures.^122^ Some HSPs are regulated by organism-specific alternative sigma factors which replace the normal regulatory σ^70^ protein of the bacterial RNA polymerase under stress conditions.^123^ The heat shock response of *E. coli* is mainly regulated by the heat shock sigma factor σ^32^ (also known as σ^H^), encoded by *rpoH*. The σ^32^ regulon includes major molecular chaperones such as DnaJ, DnaK, GroES, GroEL, HtpG and ClpB, and the nucleotide exchange factor, GrpE, which work together to refold misfolded proteins (Figure 1b). This regulon also includes ATP-dependent proteases such as ClpAP, ClpXP, Lon and FtsH which degrade irreversibly denatured proteins.^122,124^

Antibiotics that result in the misfolding or aggregation of proteins and thereby mimic heat stress have also been reported to induce the σ^32^ regulon. These include β-lactams, aminoglycosides, fluoroquinolones, trimethoprim and trimethoprim-sulfamethoxazole (Table 2). Heat-adapted bacterial strains have been reported to display increased resistance to antibiotics that mimic the effects of high temperatures, such as nitrofurantoin, trimethoprim and aminoglycosides.^125,127^ The upregulation of various heat shock chaperones and proteases, which reduce accumulation of proteins that result from exposure to aminoglycosides and fluoroquinolones, has been linked to survival in the presence of these agents.^128,133,134,136,137^

**Table 2.** Antibiotic induction of the heat shock (σ^32^) regulon and its role in antibiotic resistance

Organism^a^	Antibiotic inducer(s)	Antibiotics to which resistance conferred	Chaperones and proteases up-regulated
*A. baumannii*	Aminoglycosides, carbapenems, cephalosporins, penicillins, penicillins + BLIs, trimethoprim-sulfamethoxazole^125,126^	Aminoglycosides^125^	ClpB, DnaK, GroEL^125,126^
*E. coli*	Aminoglycosides, cephalosporins, fluoroquinolones, penicillins, tetracyclines, trimethoprim^4,50,126–131^	Aminoglycosides, fluoroquinolones, nitrofurantoin, trimethoprim^127,128^	ClpB, DnaJ, DnaK, GroEL, GroES, GrpE HtpG, HslU, HtpX, IbpA, IbpB^50,127,128,131,132^
*P. aeruginosa*	Aminoglycosides, fluoroquinolones^133,134^	Aminoglycosides, fluoroquinolones^132–134^	FtsH, HtpX, Lon^132,134^
*S.* Typhimurium	Fluoroquinolones^135^	NR	IbpB^135^

BLI, β-lactam + β-lactamase inhibitor combination; NR, not reported (to the best of our knowledge).

^a^Full organism names: *Acinetobacter baumannii*, *Escherichia coli*, *Pseudomonas aeruginosa*, *Salmonella enterica* serovar Typhimurium.

## Oxidative stress response

As many Gram-negative organisms are able to grow in the presence of oxygen, they are exposed to oxidative stress in the form of reactive oxygen species (ROS), such as superoxide radicals (O_2_•^−^), hydrogen peroxide (H_2_O_2_) and free hydroxyl radicals (•OH). These by-products of aerobic respiration damage nucleic acids, proteins and lipids and can lead to cell death.^138,139^

To protect the cell against the damage caused by ROS, various oxidative stress regulons are induced, mainly SoxRS, which senses and responds to superoxide stress^140^ and OxyR, which defends against damage caused by hydrogen peroxide.^141^ In *E. coli*, members of these regulons include superoxide dismutases encoded by *sodA*, *sodB* and *sodC*, which convert superoxide to hydrogen peroxide, and catalases encoded by *katG* and *katE*, which degrade hydrogen peroxide into water and oxygen^142^ (Figure 1c). SoxS and its homologues, MarA, RamA and Rob, are members of the AraC/ZylS family of transcriptional regulators which co-regulate overlapping genes including the AcrAB-TolC efflux pump^143,144^ and one another^145–148^ in response to oxidative stress. These oxidative stress regulons and their members, or homologues thereof, are conserved among numerous other Gram-negative bacteria.^142^

ROS production can also be stimulated by treatment with bactericidal antibiotics and there is an ongoing debate about its role in the killing action of these agents.^149–154^ SoxS, MarA and Rob and their regulon members have been shown to be up-regulated by exposure to fluoroquinolones, β-lactams, aminoglycosides, tigecycline and trimethoprim (Table 3). This was linked with increased survival in the presence of these and other antibiotic agents and is thought to be due to upregulation of the AcrAB-TolC and MdtG efflux pumps and downregulation of porins such as OmpF (Table 3).

**Table 3.** Antibiotic induction of oxidative stress responses and their role in antibiotic resistance

Regulon/response	Organism^a^	Antibiotic inducer(s)	Antibiotics to which resistance conferred	Resistance mechanisms	
				Up-regulated	Down-regulated
OxyR	*P. aeruginosa*	Aminoglycosides, polymyxins^67^	NR	NR	NR
	*V. cholerae*	Polymyxins^76^	NR	NR	NR
SoxRS	*E. coli*	Carbapenems, fluoroquinolones, penicillins^50,154–157^	Cephalosporins, chlorhexidine, fluoroquinolones, tetracyclines^155,158^	AcrAB, MdtG^156,157^	OmpF^156,157^
	*K. pneumoniae*	Fluoroquinolones^159^	Carbapenems, cephalosporins, chlorhexidine, fluoroquinolones, penicillins, tetracyclines^159^	AcrAB, TolC^159–161^	NR
	*P. aeruginosa*	Aminoglycosides, polymyxins^67^	NR	MexGHI-OpmD^67^	NR
	*S*. Typhimurium	Fluoroquinolones^162,163^	Chlorhexidine, fluoroquinolones, penicillins^162,163^	AcrAB, TolC^162^	OmpF^162^
MarRAB	*E. coli*	Carbapenems, FQs, trimethoprim^50,155–157,164^	Carbapenems, cephalosporins, chlorhexidine, fluoroquinolones, tetracyclines^155,157^	AcrAB, MdtG, TolC^156,157^	OmpF^145,156,157^
	*K. pneumoniae*	Tetracyclines^161^	Tetracyclines^144,161^	AcrAB^144,161^	NR
	*S*. Typhimurium	Fluoroquinolones^162^	Fluoroquinolones^162^	AcrAB, TolC^162^	OmpF^162^
Rob	*E. coli*	Carbapenems^157^	Carbapenems^157^	AcrAB, MdtG^156,157^	OmpF^157^
RamA	*E. cloacae*	NR	Tetracyclines^165,166^	AcrAB, OqxAB^165,166^	NR
	*K. aerogenes*	NR	Tetracyclines^166^	AcrAB^166^	NR
	*K. pneumoniae*	Cephalosporins, fluoroquinolones, tetracyclines^159,167^	Aminoglycosides, carbapenems, cephalosporins, chlorhexidine, fluoroquinolones, macrolides, monobactams, novobiocin, penicillins, polymyxins, tetracyclines^159,160,167–173^	AcrAB, LpxC, LpxL-2, LpxO, OqxAB^159,160,168–174^	OmpK35^160,171^
	*S*. typhimurium	Fluoroquinolones^162^	Carbapenems, cephalosporins, chlorhexidine, fluoroquinolones, monobactams, novobiocin, penicillins, polymyxins, tetracyclines, trimethoprim^162,163,175–177^	AcrAB, TolC^162,163,175–178^	OmpF^162,176,178^
PhoP/PhoQ	*S*. Typhimurium	NR	Macrolides^179^	MacAB-TolC^179^	NR
MexT	*P. aeruginosa*	Polymyxins^67^	NR	MexEF-OprN^67^	NR
MexZ		Aminoglycosides, chlorhexidine, macrolides, polymyxins, tetracyclines^67,180^	Aminoglycosides, fluoroquinolones, macrolides, tetracyclines^180,181^	MexXY-OprM^67,180,181^	NR
MexR and NfxB		Polymyxins^67^	NR	MexAB-OprM, MexCD-OprJ, MexJK^67^	NR

BLI, β-lactam + β-lactamase inhibitor combination; NR, not reported (to the best of our knowledge).

^a^Full organism names: *Escherichia coli*, *Klebsiella aerogenes*, *Klebsiella pneumoniae*, *Pseudomonas aeruginosa*, *Salmonella enterica* serovar Typhimurium, *Stenotrophomonas maltophila*, *Vibrio cholerae*.

RamA is found only in *Klebsiella*, *Salmonella*, *Enterobacter* and *Citrobacter* spp.^182^ and its overexpression and subsequent upregulation of components of AcrAB-TolC has been widely associated with resistance to multiple antibiotics in these organisms (Table 3). The involvement of SoxS and MarA in AcrAB-TolC-mediated resistance in this group of organisms is not well described, with some studies reporting no or lesser involvement than RamA.^144,159–163,175,183^

In addition to its effects on AcrAB-TolC, RamA has been linked with upregulation of other efflux pumps such as OqxAB and proteins involved in lipid A biosynthesis (Lpx proteins), downregulation of porins such as OmpF and OmpK35 and MDR (Table 3). These mechanisms are, however, thought to be of less importance in resistance than AcrAB-TolC. There also appears to be a consensus that activation of AcrAB-TolC by RamA or SoxS only confers low-level resistance or cannot cause resistance on its own.^159,161,168,169^ It is rather believed to be complementary to other resistance mechanisms in the development of higher-level resistance, especially upon exposure to sub-inhibitory antibiotic concentrations.^159,168^

The AcrAB-TolC homologue in *P. aeruginosa*, MexAB-OprM, as well as other Mex-type efflux pumps, respond to oxidative stress and have been reported to be induced by colistin (polymyxin E) (Table 3). Colistin was found to induce OxyR and SoxR and their regulon members, including the MDR efflux pump, MexGHI-OpmD.^67^ MexXY-OprM is induced by numerous other antibiotics, including its main substrate, aminoglycosides, resulting in resistance to these agents (Table 3).

These efflux pumps have also been described to play key roles in quorum sensing in *P. aeruginosa*.^184–188^ The *lsr* operon that regulates quorum sensing in various Gram-negative bacteria, has been reported to be a part of the SoxS regulon in *K. pneumoniae*,^189^ while the repressor of this operon, LsrR, has been shown to negatively regulate several oxidative stress response genes in *E. coli*^190^ and *S*. Typhimurium.^191^ In *E. coli*, LsrR is down-regulated in response to bactericidal antibiotics, norfloxacin, ampicillin and kanamycin,^149^ and has been linked with decreased susceptibility to a variety of quinolones as well as tetracycline, by upregulating the MFS efflux pump, MdtH.^192^

The macrolide efflux pump, MacAB-TolC, as well as its MacABCsm homologue in *Stenotrophomonas maltophilia*, has also been reported to play a role in protection against oxidative stress^193,194^ and linked to resistance to macrolides, polymyxins and aminoglycosides.^102,179,194^ However, it was not found to be involved in macrolide resistance in the case of *S. marcescens*.^194^

## SOS response

Bacteria are frequently exposed to DNA-damaging agents in the environment. These include ultraviolet (UV) irradiation,^195^ high pressure,^196^ and ROS produced during cellular respiration.^197^ Bacteria have evolved different mechanisms to repair the damaged DNA, regulated as part of the SOS response.

Upon DNA damage, the RecA protein binds to single-stranded DNA (ssDNA) and signals SOS induction. This induces the autocatalytic cleavage of the LexA repressor from the SOS box, initiating the expression of the SOS regulon genes which repair DNA lesions mainly by three pathways (Figure 1d). During homologous recombination, RecA is recruited by other homologous recombination proteins, RecJ, RecFOR and RecBCD, to repair the ssDNA lesions.^198^ The RecFOR recruits RecA to ssDNA gaps after they are enlarged by the RecJ exonuclease, while the RecBCD exonuclease/helicase complex recognizes double-stranded DNA breaks and creates an ssDNA substrate for RecA. With nucleotide excision repair, the UvrABC endonuclease proteins recognize lesions in dsDNA and cut the DNA. The UvrD helicase then removes the damaged region of DNA and DNA polymerase I (Pol I) fills the gap in an error-free manner.^199^ If the SOS-inducing signal continues, translesion synthesis occurs and this may result in mutagenesis due to the effects of the error-prone polymerases, Pol II (encoded by *polB*), Pol IV (*dinB*) and Pol V (*umuCD*).^198^ Inhibition of cell division by SulA is thought to allow time for DNA polymerases to repair damaged DNA.^200^

These SOS regulon genes are also induced upon treatment with DNA damaging antibiotics such as fluoroquinolones and metronidazole (Table 4). Fluoroquinolone treatment has been associated with upregulation of error-prone polymerases that result in mutagenesis and subsequent resistance to these agents.^201,215,216,220^ Antibiotics that do not directly result in DNA damage can also induce the SOS response. These include β-lactams, aminoglycosides, nitrofurantoin, and trimethoprim and sulfamethoxazole separately and in combination (Table 4). The SOS response has also been linked with resistance to many of these agents. For many of these bactericidal antibiotics, this DNA damage occurs indirectly due to various downstream metabolic changes, including depletion of nucleotide pools, and build-up of toxic by-products such as ROS.^164,198^

**Table 4.** Antibiotic induction of the SOS response and its role in antibiotic resistance

Organism^a^	Antibiotic inducer(s)	Antibiotics to which resistance conferred	Resistance mechanisms	
			Up-regulated	Down-regulated
*E. coli*	Carbapenems, cephalosporins, fluoroquinolones, monobactams, metronidazole, nitrofurantoin, penicillins, sulfamethoxazole and trimethoprim (separately and together)^50,164,197,201–210^	Aminoglycosides, nitrofurantoin, trimethoprim^207^	OmpX, Pol II (*polB*), Pol IV (*dinB*), Pol V (*umuCD*), QnrB, QnrD, UvrAB^164,201,211–213^	NR
*K. pneumoniae*	Aminoglycosides^b,214^	NR	NR	NR
*P. aeruginosa*	Fluoroquinolones^215,216^	Fluoroquinolones^216^	CTX-M-27, DnaE2, ImuB, UmuD, various variants of OXA^215–218^	NR
*S. marcescens*	Fluoroquinolones^c,212^	Fluoroquinolones^c,219^	SmaQnr^212,219^	NR
*S*. Typhimurium	Fluoroquinolones^220^	NR	UvrA, Pol II (*polB*)^220^	OmpA, OmpD, various *flg and fli* genes^220^
*V. cholerae*	Aminoglycosides, chlorhexidine, fluoroquinolones, rifamycins, tetracyclines, trimethoprim^202,205,214^	NR	NR	NR

NR, not reported (to the best of our knowledge).

^a^Full organism names: *Escherichia coli*, *Klebsiella pneumoniae*, *Pseudomonas aeruginosa*, *Salmonella enterica* serovar Typhimurium, *Serratia marcescens*, *Vibrio cholerae*.

^b^Induction of SOS-regulated gene, no specific SOS-regulated resistance mechanism tested.

^c^Smaqnr of *S. marcescens* cloned into *E. coli*.

In *E. coli*, β-lactams were also found to induce the SOS response via upregulation of the DpiBA TCS, which interrupts DNA replication and thereby induces the SOS response.^202^ LexA binding sites (SOS boxes) have been identified upstream of some plasmid-mediated resistance genes, such as the *bla*_CTX-M_ and *bla*_OXA_ β-lactamase genes^217,218^ and the *qnrB* quinolone resistance gene.^211,221^ Expression of these resistance genes can therefore also be up-regulated by the SOS response in response to treatment with bactericidal antibiotics.^211,212,218,221,222^

The SOS response also promotes HGT of antibiotic resistance genes.^217,223,224^ The exact mechanism of this SOS-mediated transfer is unclear, but it is thought to be through the action of RecA. This could also be through the induction of OMVs which are involved in HGT, as the SOS response has been reported to be involved in stimulation of OMVs in *P. aeruginosa*.^92^ This may be through induction of SulA, which inhibits cell division during DNA repair and may temporarily affect the structure of the cell envelope and stimulate OMV production.^225^

## General stress response

When exposed to stressors of various kinds in their natural environments, bacteria slow down their growth and use their limited resources only for stress survival and maintenance. This is known as the stationary phase response or general stress response. In *E. coli* and related Gram-negative bacteria, this response is regulated by the RpoS (σ^S^ or σ^38^) regulon, which includes more than 500 genes.^3^ The general stress response not only supports slow or stationary phase growth but is also rapidly induced in parallel to specific stress responses as an emergency survival mechanism when exponentially growing cells are suddenly exposed to potentially lethal stress conditions (Figure 1e).^3^ If the specific responses are sufficient to combat the stressor, the general stress response is switched off. Many genes regulated by specific stress responses are therefore co-regulated by RpoS.

β-Lactams, aminoglycosides and quinolones have been reported to induce the RpoS regulon and RpoS has been linked to survival in the presence of carbapenems, quinolones, PMB, rifampicin and tetracycline (Table 5). Other than these, very few studies report on antibiotic induction of RpoS or its role in antibiotic resistance. Antibiotic survival may be attributed to decreased expression of the OmpF porin, which has been shown to be regulated by RpoS under nitrogen limitation.^231^ While some studies have shown that RpoS induction (together with SOS induction) is required for upregulation of Pol II and Pol IV expression, there are contrasting findings regarding the involvement of RpoS in mutagenesis.^226,228–230,235,236^

**Table 5.** Antibiotic induction of the general stress response (σ^S^) regulon and its role in antibiotic resistance

Organism^a^	Induced by	Antibiotics to which survival is enhanced	Resistance mechanisms	
			Up-regulated	Down-regulated
*E. coli*	Cephalosporins, fluoroquinolones, penicillins^226^	Fluoroquinolones, rifamycins, tetracyclines^226,227^	Pol II (*polB*), Pol IV (*dinB*)^228–230^	MutS, OmpF^226,231^
*P. aeruginosa*	NR	Carbapenems, fosfomycin, fluoroquinolones, polymyxins^226,232–234^	NR	MutS^226^
*V. cholerae*	NR	Rifamycins^226^	NR	MutS^226^

NR, not reported (to the best of our knowledge).

^a^Full organism names: *Escherichia coli*, *Pseudomonas aeruginosa*, *Vibrio cholerae*.

One suggestion is that the mutagenic activity of Pol IV is regulated directly by RpoS during stationary phase but indirectly during exponential phase.^228^ This indirect regulation is mediated by decreased production of both ClpXP protease, which degrades Pol IV and σ^S^ in the absence of stress,^236,237^ and MutS, which is involved in mismatch repair to correct the errors of Pol IV.^226^ It is important to remember that the general stress response acts mainly as an emergency reserve stress response and, therefore, likely contributes to the development of antibiotic resistance through aiding the specific stress responses, rather than directly. For example, RpoS was shown to play a vital role in induction of the SOS response upon aminoglycoside exposure and subsequent oxidative stress.^214^

## Interactions between stress responses

Bacterial stress responses act as a complex interconnected network and many stress response proteins involved in antibiotic survival have been shown to be regulated by more than one stress response. Various ESRs have been found to be dependent on each other for their own activation or for collaborative regulation of proteins involved in antibiotic resistance.^19,21,22,24,25,43,52^

The envelope stress regulons additionally respond to oxidative stress^9,69,102,238–240^ and heat shock,^116,136,137,241–244^ likely because these stressors all result in protein misfolding and membrane damage. Many of these respective stress response regulon members, such as porins and efflux pumps, are therefore co-regulated amongst each other.^11,21,24,25,47,245,246^ In this way, damage affecting different compartments of the cell can be alleviated, for example, with heat shock regulons regulated by σ^32^ in response to intracellular protein damage and σ^E^ in response to extracytoplasmic stress.^128,137,238,245^

Furthermore, the periplasmic degradation protease, DegP (also known as HtrA), which is regulated by more than one stress response regulon, is able to switch functions when exposed to different stress conditions. It has been reported to act as a chaperone at normal temperatures or during oxidative stress and as a protease at higher temperatures.^247,248^ Some heat shock chaperones have also been shown to (either directly or indirectly) interact with certain SOS regulon members, such as Pol IV and Pol V, and aid in their folding.^249–251^ After DNA damage has been repaired by the SOS response, SulA is degraded by the Lon and HslUV heat shock proteases to allow cell division to continue.^252,253^

## Targeting stress responses to reduce antibiotic resistance: emerging therapeutic strategies and knowledge gaps

The increasing rate of antibiotic resistance is threatening the effectiveness of current antibiotics to treat serious infections, and overcoming this global health crisis is becoming a matter of urgency. Since various antibiotic-induced stress responses have been linked to antibiotic resistance, as summarized through this review, they are increasingly being considered as promising targets for the development of therapeutics to enhance the bactericidal activity of antimicrobial agents.^203,254,255^

A common thread seen throughout this review is the upregulation of efflux pumps by various stress responses. Efflux pump inhibitor compounds able to reduce antibiotic resistance have been reported since the early 2000s.^256–259^ For example, phenylalanylarginine β-naphthylamide (PAβN) and 1-(1-naphthylmethyl)-piperazine, were demonstrated to inhibit AcrAB and AcrEF efflux pumps and decrease fluoroquinolone resistance.^257,259,260^ These compounds have potential as antibiotic adjuvants and efflux pumps remain promising targets for future drug discovery efforts.

Various compounds able to inhibit the SOS response have been identified, such as zinc,^224^ curcumin^261^ and *N*-acetylcysteine (NAC).^197^ Among these, NAC has been demonstrated to reduce SOS-mediated mutagenesis induced by ciprofloxacin^197^ and zinc has been shown to inhibit the SOS-induced horizontal transfer of resistance genes.^217^

Furthermore, disruption of levofloxacin-induced heat shock genes, *dnaK*, *groEL* and *lon*, was shown to increase susceptibility to this agent, with the *lon* mutant displaying the greatest effects.^128^ It was suggested that agents capable of inhibiting the Lon protease have the potential to be used as combination therapy with fluoroquinolones. Peptidic boronic acid inhibitors have been presented as potent inhibitors of Lon in *E. coli* and *S. enterica* serovar Typhimurium and linked with a reduction in ciprofloxacin tolerance.^262,263^

However, inactivation of *lon* has been shown to cause low-level MDR in *E. coli* by stabilizing MarA and SoxS, which are substrates of the Lon protease, and subsequently inducing the AcrAB-TolC pump.^264^ In addition to the heat shock response, the Lon protease is involved in the SOS,^253^ oxidative stress^265^ and ESRs.^243^ Its inhibition might, therefore, affect other stress responses and influence antimicrobial susceptibility in various ways.

This, together with the compensatory nature of stress responses seen throughout this review, emphasizes the notion that future studies should take a holistic approach when investigating stress responses. This could mean moving away from single gene knockouts and a shift towards more research on stress response inhibitor compounds that can target multiple members of a particular stress response or more than one response. Similarly, the use of more global transcriptomic approaches such as RNA-sequencing can overcome the limitations of targeted methods such as green-fluorescent protein induction, reverse-transcription quantitative real-time PCR and microarrays commonly employed by studies in this review.^266,267^ This would enable a more holistic investigation of the global responses and compensatory mechanisms influenced by inhibition of individual stress responses.

RNA-seq has been used to investigate the global response to antibiotics in *E. coli*, *S*. Typhimurium and *A. baumannii.*^204,220,268–271^ However, more research on this topic should include other Gram-negative ESKAPE pathogens such as *K. pneumoniae* and *P. aeruginosa*, as well as antibiotic-resistant pathogens on the WHO bacterial priority pathogens list^272^ to aid in the fight against these dangerous organisms.

Additionally, use of experimental conditions that mimic the infected host environment could provide a better understanding of these organisms in their ‘natural habitats’. For example, bacterial metabolism in laboratory culture media is different to that in a host and differential gene expression of *E. coli* cultured in human clinical samples compared with Luria Bertani medium has been demonstrated.^273^ Metatranscriptomic approaches using RNA extracted from whole clinical specimens before and after commencing antibiotic treatment or culturing of isolates in bacteria-free specimens could be considered in future studies.

In addition to the specific bacterial genotype, interactions between bacteria in populations and interspecies communities can affect their stress responses.^274,275^ These competitive and cooperative interactions play a role in the susceptibility of community members to antimicrobials and facilitate the development and spread of antibiotic resistance.^276–278^ Studies employing bacterial co-culture approaches would be a good starting point to investigate the effects of intra- and interspecies interactions on bacterial stress responses and their role in resistance, especially via HGT. In the longer term, biological matrices with more complex mixed bacterial populations, such as stool samples, could be used to assess stress responses and subsequent development of antibiotic resistance. This could inform strategies to reduce the selection of antibiotic resistance in commensal gut microbiota during antibiotic treatment.

## Conclusions

For the most part, there are clear correlations between the mechanisms of action of antibiotics and the stress responses they induce, such as β-lactams which target the cell wall and induce ESRs, or fluoroquinolones which result in DNA damage and induce the SOS response. Many of these stress responses are conserved among Gram-negative bacteria or occur via inter-species homologues. However, we have noted that some survival mechanisms can be species-specific. Some studies have focused on the Gram-negative ESKAPE pathogens, such as *K. pneumoniae*, *A. baumannii*, *P. aeruginosa*, and *Enterobacter* spp., and further explorations into the stress response networks of these infamous MDR organisms could be beneficial for targeted approaches in combating antibiotic resistance.

Antibiotics may induce more than one stress response, as a result of indirect effects other than those on the common target site. Stress responses are vastly interconnected, and none seem to be sufficient to promote survival in the presence of antibiotic stress on their own. Instead, they work together as a highly co-ordinated network, where decreased expression of one member can result in increased expression of another to compensate and promote survival. Bacteria clearly have many ‘backup’ options in case one stress response is not completely successful in combating the stress, or if damage repair needs to occur at different components of the cell simultaneously.

Studies have suggested that agents capable of inhibiting certain stress response proteins could be considered as combination therapies with antibiotics to increase their activity and reduce the development of resistance. This review provides an overview of possible stress response targets for future research directed towards this promising avenue of fighting antibiotic resistance.
